# A Comparison Between Saline and Balanced Solutions in Kidney Transplants: A Randomized Clinical Trial

**DOI:** 10.7759/cureus.49813

**Published:** 2023-12-01

**Authors:** Heitor Medeiros, Paulo H Lima, Vital S Junior, Diego A Souza, Aline M Pinheiro, Rand R Martins, Kellen M A H Costa, Jose Hipolito D Junior, Paulo J Medeiros, Wallace A Da Silva

**Affiliations:** 1 Department of Anesthesiology, Hospital Universitário Onofre Lopes, Natal, BRA; 2 Graduate Program of Pharmaceutical Assistance, Universidade Federal do Rio Grande do Norte, Natal, BRA; 3 Department of Nephrology, Universidade Federal do Rio Grande do Norte, Natal, BRA; 4 Department of Urology, Hospital Universitário Onofre Lopes, Natal, BRA

**Keywords:** acidosis, plasmalytes, balanced solutions, balanced crystalloids, • kidney transplantation

## Abstract

Objective

This study aimed to investigate the impact of different types of intravenous fluids - normal saline (NS), lactated Ringer’s solution (LR), and PlasmaLyte (PL) - on the acid-base balance and electrolyte concentration following kidney transplant, a common procedure for patients with end-stage renal disease (ESRD).

Methodology

A randomized controlled trial design was employed, wherein the primary parameters analyzed were postoperative pH and serum potassium levels. Postoperative concentrations of serum bicarbonate, sodium, chloride, and creatinine, as well as graft functionality, were assessed as secondary outcomes. These measurements were performed at the start and end of surgery, as well as 24 and 72 hours postoperatively.

Results

A total of 53 patients were included in the study and randomized into three cohorts: NS, LR, and PL, each of which showed comparability in terms of demographic and transplantation specifics. Notably, patients in the NS group exhibited a more significant decrease in pH (NS group: 7.285 ±0.098, LR group: 7.324 ±0.075, PL group: 7.7338 ±0.059) and bicarbonate levels (17.0 ±4.2, 20.9 ±2.8, 20.0 ±4.5) post 24 hours after the operation and displayed a similar pattern immediately after the surgery. However, there were no discernible differences in potassium (p=0.460), sodium (p=0.681), and chloride (p=0.321) levels across the groups. Furthermore, the study did not observe any significant differences in postoperative graft functionality.

Conclusion

The use of NS as the intraoperative fluid of choice led to lower pH and bicarbonate levels following kidney transplant, as compared to LR and PL. However, these results did not correlate with improvements in graft functionality.

## Introduction

Kidney transplant is the treatment of choice for patients with end-stage renal disease (ESRD) and presents several advantages over dialysis, including better long-term survival [1]. The success of kidney transplants relies on several aspects related to recipient care, including maintenance of acid-base homeostasis and intravascular volume [2-4]. However, the best practice regarding the choice of intravenous fluid in this scenario remains unclear. Patients undergoing kidney transplants commonly present with metabolic disturbances such as acidosis and hyperkalemia. Although normal saline (NS) is widely used due to its lack of potassium, the administration of this crystalloid can lead to metabolic acidosis with subsequent hyperkalemia due to extracellular shifts in potassium [5]. Moreover, the infusion of hyperchloremic solutions can cause renal vasoconstriction, leading to kidney injury [6,7].

Balanced crystalloids, such as lactated Ringer’s (LR) and PlasmaLyte (PL), are closer to plasma in composition and have increasingly been used as alternatives to NS in various scenarios. Although these fluids contain physiological concentrations of potassium, they are believed to prevent hyperkalemia owing to their alkalizing properties [7,8]. In light of the ongoing debate as to which crystalloid is the most appropriate in patients undergoing kidney transplants, we conducted a randomized controlled trial to compare the effects of intraoperative NS, LR, and PL on acid-base status and electrolyte concentration.

## Materials and methods

The ethical approval for this study was obtained from the Bioethics Committee of the Medical University of Hospital Universitario Onofre Lopes (CAAE 97769618.0.0000.5292); all procedures performed in this study involving human participants were in accordance with the ethical standards of the institutional research committee and adhered to the 1964 Helsinki Declaration and its later amendments or comparable ethical standards. We enrolled patients diagnosed with ESRD who received a transplant from a living or cadaveric donor between December 2020 and December 2021. The exclusion criteria included patient refusal, age under 18 years, serum potassium level higher than 6.5 mEq/L at admission, and previous kidney transplant. After obtaining written informed consent, patients were randomized into three groups using sealed envelopes based on the type of crystalloid used intraoperatively: NS, LR, or PL. All eligible patients were included in the randomization procedure. Allocation numbers were derived from a random number table by an assistant other than the one who enrolled the patient into the intervention or control group and were placed in envelopes that were opened only by the anesthesiologist who performed the intervention.

Anesthesia was performed according to the institutional protocol [8]. Standard monitoring with electrocardiography, noninvasive blood pressure measurement, pulse oximetry, nasopharyngeal thermometry, and capnography was done for all patients. In high-risk cases, arterial catheterization for invasive monitoring and a central line were established. General anesthesia was induced with propofol, fentanyl, and cisatracurium, and sevoflurane was used for maintenance. Analgesia was provided through the administration of opioids or a combination of general anesthesia with a spinal or peripheral block [ultrasound-guided transversus abdominis plane (TAP) block]. The hemodynamic management aimed to provide the optimal mean arterial pressure (MAP) throughout the procedure, particularly during kidney reperfusion. Mannitol and furosemide were administered intraoperatively to improve kidney reperfusion and urinary output. The study fluid was discontinued at the end of the surgery, and fluid management was performed by the nephrology staff. Because the bags containing crystalloids were different in shape, blinding of the personnel was not possible.

The primary outcomes were postoperative pH and serum potassium levels. Secondary outcomes included postoperative concentrations of serum bicarbonate, sodium, chloride, and creatinine. Electrolyte measurements and acid-base status were evaluated using venous blood samples at the beginning of the surgery, end of the surgery, and 24 and 72 hours after the surgery. Hyperkalemia was treated according to the institutional protocol when the serum potassium level was 6.5 mEq/L or higher. There were no changes in trial outcomes definitions after the trial commenced. MAP and urinary output (UO) were recorded at different times. Graft function was analyzed by measuring serum creatinine and UO levels at 24 and 72 hours after the procedure. The need for dialysis and treatment of hyperkalemia within 72 hours was also documented. Additionally, we assessed the length of hospital stay and in-hospital mortality rate. All laboratory analyses were performed at our institution’s central laboratory.

The necessary sample size was calculated based on previous studies, with a view to detecting an intergroup difference of 0.1 in units of pH [standard deviation (SD) of 0.07] and a difference of 1 mEq/L of potassium (standard deviation of 0.6), two-sided alpha of 5%, and power of 80% [9,10]. We aimed to enroll at least 15 patients in each group to allow for a 20% dropout rate. Data were assessed for normality by using the Shapiro-Wilk test. Categorical data were analyzed using Pearson’s chi-squared test or Fisher’s exact test, whereas quantitative data were assessed using ANOVA or the Kruskal-Wallis test. A linear mixed-effects model was used to compare variations in pH, bicarbonate, and electrolytes between the groups over time. Categorical variables are reported as frequencies, whereas continuous variables are reported as means and SD. Data were analyzed using the JASP software (JASP Team, 2018), and statistical significance was set at p<0.05.

## Results

**Figure 1:**
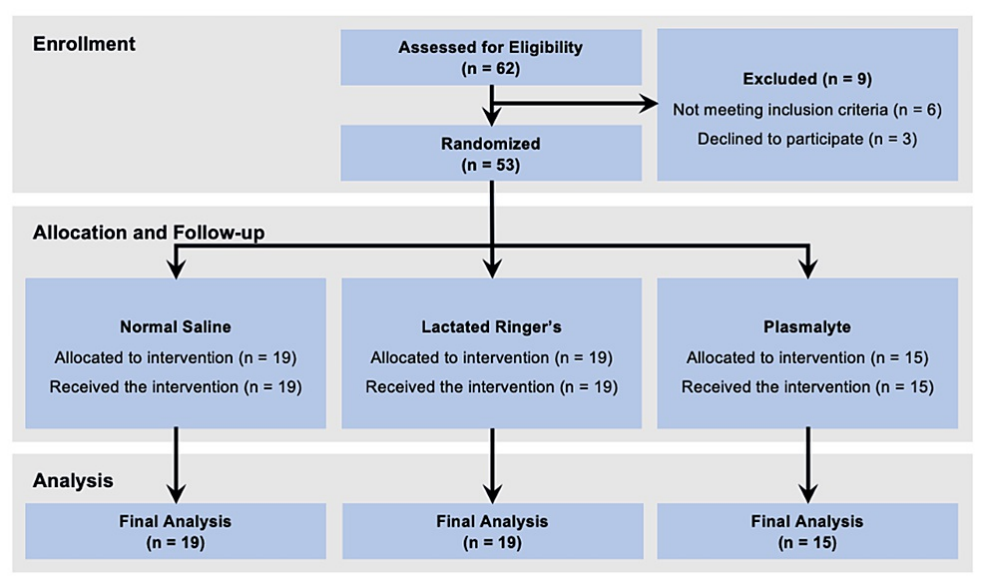
Flow chart depicting the selection of the study population

**Figure 2:**
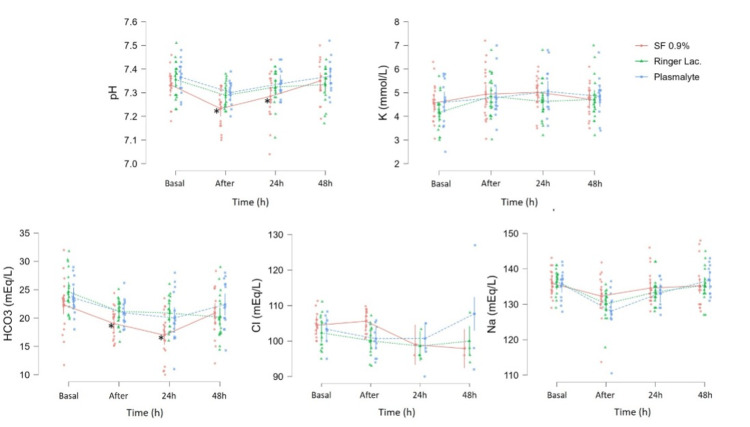
The linear mixed-effect model of pH, bicarbonate, and electrolytes variations between groups over time *Significant statistical difference between normal saline group versus lactated Ringer’s and Plasmalyte groups Values are presented in mean ±SD SD: standard deviation

Between December 2020 and December 2021, 62 patients underwent renal transplantations at our institution. Nine patients were not included based on the exclusion criteria. A total of 53 patients were included in the study: 19 were randomized to the NS group, 19 to the LR group, and 15 to the PL group, and none of them were lost to follow-up (Figure 1). The patient demographics and transplantation variables are presented in Table 1. Figure 2 illustrates the variations in the pH and electrolyte concentrations between the groups over time. Once the necessary sample size was attained, we went on to include eight extra patients (a total of 53) to account for possible missing data or unexpected dropouts.

**Table 1:** Patient demographics and transplant-related variables CIT: cold ischemic time; LR: lactated Ringer’s; MAP: mean arterial pressure; NS: normal saline; PL: PlasmaLyte; RBC: red blood cells; SD: standard deviation

Characteristic	NS (n=19)	LR (n=19)	PL (n=15)	P-value
Age, years, mean (SD)	48.2 (14.9)	41.4 (10.4)	49.1 (10.3)	0.137
Male gender, n (%)	10 (52.6)	9 (47.4)	10 (66.7)	0.519
Actual weight, kg, mean (SD)	63.5 (9.1)	67.9 (18.7)	62.6 (16)	0.613
Donor, cadaveric, n (%)	17 (89.5)	18 (94.7)	14 (93.3)	0.819
CIT, hours, mean (SD)	10.8 (5.9)	12.6 (5.5)	16 (11)	0.198
Anesthetic technique, combined, n (%)	11 (57.9)	14 (73.7)	9 (60)	0.552
Intraoperative volume, mL, mean (SD)	2933.3 (897.1)	2406.7 (536.5)	2418.2 (365.6)	0.055
Need for RBC transfusion, n (%)	4 (25)	1 (5.3)	1 (7.7)	0.193
Use of norepinephrine, n (%)	2 (11.1)	1 (5.3)	3 (20)	0.409
Intraoperative MAP, mmHg, mean (SD)				
Initial	95.6 (16.9)	101 (22.9)	98.7 (27.4)	0.769
Pre-reperfusion	77.2 (8.1)	77.3 (10.9)	79.1 (10.7)	0.829
Post-reperfusion	90.4 (6.1)	91.8 (7.5)	91.8 (10.1)	0.837
Final	94.2 (12.4)	98 (16.2)	104.2 (10.9)	0.184
Dose of mannitol, mL, mean (SD)	216.9 (48.2)	222.4 (50.6)	217.9 (50.4)	0.940
Dose of furosemide, mg, mean (SD)	71.4 (23.9)	68.4 (30.2)	69.3 (23.7)	0.941

Patient demographics and transplant-related variables

All the groups were comparable in terms of age, sex, and weight. Furthermore, there were no significant differences in the origin of the graft (living or cadaveric donor) or cold ischemia time. Combined anesthesia was the preferred anesthetic technique. Despite the fact the mean volume of crystalloid infused in the NS group was somewhat higher (2933.3 mL, versus 2406.7 mL in the LR group and 2418.2 mL in the PL group), this difference was not statistically significant. The need for RBC transfusion did not present a statistically significant difference between groups. Intraoperative red blood cell transfusion and use of norepinephrine were required in six patients (no statistical difference between groups). However, the MAP measured at different times of surgery was comparable, indicating that macrohemodynamic control did not vary among the groups. Intraoperative doses of mannitol and furosemide were also similar.

Acid-base status and electrolytes concentration analyses

At the beginning of surgery, the pH values did not vary between the groups (p=0.398). The same was true for the serum concentrations of bicarbonate (p=0.213), potassium (p=0.213), sodium (p=0.846), and chloride (p=0.416). The means and standard deviations of electrolyte concentrations and pH values at different time points are presented in Table 2. To test the difference between the means, ANOVA (p<0.05) was used (Table 2).

**Table 2:** Postoperative graft function LR: lactated Ringer’s; NS: normal saline; PL: PlasmaLyte; SD: standard deviation; UO: urinary output

Characteristic	NS (n=19)	LR (n=19)	PL (n=15)	P-value
UO, mL, mean (SD)				
At the end of the surgery	127.9 (254.4)	212.6 (244.9)	167.5 (229.2)	0.602
After 24 hours	474.2 (778.5)	687.7 (739.8)	608.4 (575.1)	0.674
After 72 hours	676.5 (855.5)	1147.3 (831.5)	1135.6 (854.2)	0.179
Serum creatinine, mg/dL, mean (SD)				
At the beginning of the surgery	9.8 (2.5)	8.4 (3.5)	8.3 (3.3)	0.379
At the end of the surgery	7.9 (2.9)	8.1 (2.1)	9 (1.9)	0.829
After 24 hours	7.3 (1.9)	7.7 (2.2)	7.1 (2.7)	0.781
After 72 hours	7.2 (2.5)	7.4 (3)	7.1 (2.8)	0.962
Need for hemodialysis <72 hours, n (%)	10 (52.6)	5 (26.3)	5 (33.3)	0.226
Treatment for hyperkalemia for <72 hours,n (%)	4 (25)	4 (22.2)	1 (7.7)	0.457
Hospital stay, days, mean (SD)	22.1 (11.7)	21 (18.3)	33.7 (24.7)	0.158
In-hospital mortality, n (%)	0 (0)	1 (5.9)	0 (0)	0.505

Following transplantation, patients experienced a decrease in pH, with a posterior increase, and return to basal levels. This trend was statistically significant in all the groups (p<0.001). However, the decline was more pronounced in the NS group, both in the immediate postoperative period and after 24 hours (p=0.012). Bicarbonate analysis revealed a similar tendency. Its concentration decreased in all groups immediately and 24 hours after the surgery (p<0.001); however, patients in the NS group were more affected than those in the LR or PL groups (p=0.026).

The serum potassium concentration increased in all groups (p=0.004) and remained constant in the following 72 hours. No significant differences were observed between the groups in this regard (p=0.460). Similarly, there were no differences in sodium (p=0.681) and chloride (p=0.321) concentrations over time among the groups.

Postoperative graft function

Graft function surrogates were evaluated as secondary outcomes. No statistically significant results were found regarding UO, serum creatinine level, need for hemodialysis, or clinical treatment for hyperkalemia in the first 72 hours following transplantation. Likewise, the length of hospital stay and incidence of in-hospital mortality did not differ between the groups (Table 2).

## Discussion

In this prospective, single-center, randomized controlled study, we compared the effects of intraoperative NS, LR, and PL on the acid-base status and electrolyte concentration following kidney transplant. We found that participants who received LR and PL during kidney transplants had a better acid-base profile up to 24 hours after surgery. This finding is corroborated by previous studies that compared NS with balanced crystalloids [4,6,11-14]. The administration of NS can lead to metabolic acidosis due to the dilution of the endogenous bicarbonate buffer system, an entity known as dilutional acidosis, although this phenomenon is reported as likely not harmful and self-correcting [15]. Another explanation pertains to the Stewart model for acid-base balance. According to this model, NS has a strong ion difference (SID) of zero because sodium and chloride have equal concentrations in this crystalloid. Thus, infusion of NS decreases the SID of plasma and causes acidosis [16].

In line with the findings of a previous Cochrane meta-analysis, our study did not show any differences in serum potassium or the need for treatment for hyperkalemia for those who received balanced solutions compared to NS [9]. In the early 2000s, up to 80% of patients undergoing kidney transplantation in the US received NS as infusion therapy [17]. Since then, evidence on the safety of potassium-containing solutions in patients with impaired kidney function has been published [7]. However, NS continues to be favored over balanced crystalloids in some centers due to the lack of potassium. Actually, since NS leads to acidosis, it may exacerbate hyperkalemia due to the shift of potassium from intracellular to blood [5].

Although we did not find a positive impact of balanced crystalloids on graft function, it has been shown that the maintenance of a stable acid-base milieu following kidney transplant is related to better outcomes [18]. To date, no study has clearly stated that the administration of balanced crystalloids would positively affect graft function when compared to NS. Weinberg et al [14] performed a study where the trial fluid was continued to be administered for 48 hours after surgery. They found a trend towards a faster reduction in creatinine levels and better glomerular filtration rate in patients receiving PL. However, O’Malley et al. [12], Hadimioglu et al. [4], Kim et al. [19], and Potura et al. [6] did not obtain similar results. The fact that our study protocol was limited to the intraoperative period and was not powered to detect differences in such outcomes precludes any conclusions on this subject.

In our study, both pH and bicarbonate concentrations were similar when LR was compared with PL. These findings are similar to those obtained by Hadimioglu et al. [4] and Weinberg et al. [14]. One of the reasons could be that PL has a higher concentration of buffer anions than LR (23 mEq/L of gluconate and 27 mEq/L of acetate in PL versus 28 mEq/L of lactate in LR), making it more alkalinizing. However, several studies have reported that most of the gluconate is not metabolized in bicarbonate and has no effect on pH [20-22]. Thus, although PL has a theoretical advantage over LR, it is probably not clinically relevant. LR is moderately hypo-osmolar, and the accumulation of lactate from LR may increase serum lactate levels and lead to misinterpretation. However, LR is cheaper than PL and could be an adequate alternative to NS in low-income settings.

Another advantage of balanced crystalloids over NS is their lower chloride concentration [4,12,13]. In our study, the NS group showed a tendency toward higher plasma chloride concentration at the end of surgery; however, this finding was not statistically significant. Despite some controversies, hyperchloremia has been linked to acute kidney injury secondary to reduced blood flow to the renal cortex [23]. The administration of fluids with high chloride content should be avoided in at-risk patients, such as those undergoing kidney transplantation.

This study has some limitations. The bags of crystalloids differed in shape, forcing us to perform an open-label study. Despite this, the participants were already under general anesthesia when the infusion was started, and the researcher who performed the statistical analyses was blinded. Another limitation is that the study protocol was applied only during the surgery. Thereafter, fluid therapy was administered by the nephrology team, who generally preferred NS - and this may impact the interpretation of results and may have limited the postoperative findings. The sample size may pose challenges in terms of generalizability and statistical power. No potential harm was detected in terms of our study design.

## Conclusions

Based on our findings, NS infusion led to lower pH and bicarbonate concentrations after kidney transplant when compared with LR and PL. However, these findings were not associated with better graft function. No differences were found in potassium concentration or the need for treatment for hyperkalemia, indicating that the fear of using balanced crystalloids due to their potassium content is unjustified. Additionally, our findings suggest that LR and PL can be used as alternatives to NS without causing any significant electrolyte abnormalities. These results can contribute to improving fluid therapy in this specific scenario. We recommend further studies with larger sample sizes and a strict protocol controlling the type and amount of fluid in the postoperative period to evaluate whether balanced crystalloids improve the outcomes of kidney transplants.
